# Physical activity promotes well-being: medical students’ engagement and perspective. A cross-sectional study scoped through innovation and technology

**DOI:** 10.1097/MS9.0000000000002808

**Published:** 2025-01-09

**Authors:** Sami Mohamed, Surajo Abdulqadir Muhammad, Abdikarim Abdi Mohamed, Ahmad Al-Mujtaba Esam Eldeen, Ahmad Mahmud Alhaj, Ahmad Abdullahi Bulama

**Affiliations:** a Department of Clinical Sciences, Dubai Medical University, Dubai, United Arab Emirates; b Community Medicine Department, Nile University, Khartoum, Sudan

**Keywords:** exercise prescription, non-communicable disease prevention, physical activity promotion, technology-based PA

## Abstract

**Background:**

The role of physical activity (PA) in health promotion is further complimented by its disease preventive value. Future doctors’ involvement and advocacy in diverse aspects of such significant role is still under evaluation.

**Objectives:**

This study aimed to assess medical students’ overall PA engagement and their perspective, and to identify whether their level of engagement affects their advocacy and promotion, while discussing related educational innovations and technological advances.

**Methods:**

Through a cross-sectional observational study design, stratified sampling from third-, fourth-, and fifth-year medical students attending Nile University, Sudan, during the period from February to April 2020, were included. Descriptive data analysis was done using the SPSS software, and presented including, frequencies, cross-tabulation, Chi-square testing with 95% confidence level, and a *P* value, after data collection via a structured questionnaire adopting parts of the IPAQ. Study has been reported in line with the STROCSS criteria.

**Results:**

Out of 188 students, 43% males and 57% females, less than half (91, 48.40%) participated in planned PA, and majority (156, 82.98%) agreed with its health-related significance. Few (57, 30.32%) considered academic load as a barrier, and only 20 (10.60%) were not actively promoting PA engagement to others. Most PA participants were males (57.14%), leaning toward group-based activity (54, 59.34%), making regular plans (51, 56.04%) and motivated by self-health promotion (86, 94.51%) (*P* < 0.05). Despite majority of students (82.98%) agreement on PA’s benefits, nearly half (51.60%) do not engage, Similarly, overall advocacy for PA promotion in relation to specific people was not statistically significant (*P* = 0.21). Embedding PA into undergraduate curriculum, use of PA report cards, exercise prescription training and targeted mentoring, in addition to digital bracelets, smart phone applications and internet-based social media, had positive impact on PA participation and promotion.

**Conclusion:**

Medical students’ understanding of PA health related significance did not equate to increase engagement, despite active promotion. Curricular integration of PA should accompany recent innovative educational strategies to increase their participation and advocation. Future studies are needed to evaluate the role of technology-based and AI-driven PA in achieving this goal.

## Introduction

The beneficiary significance of physical activity (PA) within the health incorporated landscape is an established phenomenon ^[1]^. Its systematic implementation positively influences general well-being and promotes mental health^[2,3]^, while its absence acts as an attainably modifiable risk factor for development of chronic medical conditions^[4,5]^.

**Highlights**

- Students have an acceptable understanding, and advocacy, toward the significance of physical activity.
- Lack of physical activity among medical students is not related to their academic responsibilities.
- Integration of physical activity into undergraduate medical education requires innovative strategies.
- Technology-based and AI-driven physical activity may potentially enhance students’ engagement.

PA also plays a quintessential role in prevention of non-communicable diseases^[6]^, which still poses a substantial burden, regularly reported by the World Health Organization (WHO)^[7]^, particularly in low income countries^[8]^. This has led to the development of targeted practice guidelines, such as the PA Guidelines for Americans^[9]^, as well as evaluation instruments, such as the International PA Questionnaire (IPAQ)^[10]^, in order to optimally achieve its intended benefits through application, assessment and research.

PA is defined as any energy consuming bodily movement that utilizes skeletal muscles. Both moderate-intensity and vigorous-intensity improve health, including leisure time, as well as transport displacement, movement^[1]^. According to the 2020 World Health Organization Guidelines on PA and Sedentary Behavior recommendations, all adults should engage in a weekly minimum of 150 minutes moderate, or 75 minutes vigorous, intensity PA, or in an equivalent combination, in order to achieve this goal^[11]^.

In the realm of medical education, participation of undergraduate medical students in PA was extensively studied internationally^[12-24]^ Yet scientists and medical educators are still assessing various aspects pertaining to their involvement, understanding and active promotion, aiming toward improvement at all domains^[25-41]^. This sheds light on the additional importance related to future doctor’s role in PA directed healthcare promotion, which remains suboptimal^[42-44]^. Moreover, studying the overall level of PA engagement and its potential effects on students’ advocacy and PA promotion to others, may help better understand the varying discrepancy of participation between different populations.

Furthermore, the rising coronavirus disease (COVID-19) pandemic indiscriminately impacted the general population’s engagement in PA, as well as the educational process, globally affecting medical institutions^[45]^.

However, emerging technological advances including online distant learning and evaluation platforms, e-learning resources, digital simulation-based education, virtual reality technology (VR) and artificial intelligence (AI), have grown as a necessity, advocating for continuity of health professions education during dire circumstances, that is inclusive of limited resource countries^[46-48]^.

Innovative introduction of PA in curricular development within medical institutions was suggested in recent years, whether as an embedded part of undergraduate curriculum^[49-53]^, advocated by Public Health England, or by incorporating PA counseling and prescription into medical schools, advocated by Medical Council of Canada^[54,55]^.

However, discussing technological advances tailored toward PA, such as monitoring devices, wearable activity trackers, mobile technology, AI-driven recommendation systems and chatbots, as a potential influencer of medical students’ involvement and active promotion, is yet to be demonstrated.

This study aims, therefore, to assess medical students’ overall engagement in any form of PA; their motivation; their overall perspective on its preventive significance and potential barriers including academic load, while discussing the potential role of innovative educational strategies, incorporating technology-driven PA, in enhancing their adherence and advocation.

## Methods

### Study design

This was an observational, cross-sectional retrospective study conducted at Nile University, located in Khartoum, Sudan. Study population consisted of all medical students attending faculty of medicine at all grade levels extending from first to sixth year and was set as inclusion criteria. The university is open for both national and international student. Exclusion criteria was set only for those who did not consent to or were not available to participate. The work has been reported in line with the STROCSS criteria^[56]^. The study was registered at www.researchregistry.com, UIN researchregistry10728.

### Sampling

Utilizing a stratified sampling technique, participants were selected from official university listings, include all students enrolled and attending first through sixth year during the period from February to April 2020. However, only those from third, fourth, and fifth years were included. First- and second-year students were on leave and were not regularly attending the university at the time of the study, as they had a relatively different calendar timetable, and sixth-year students were occupied by ongoing end term exams. Therefore, they were not included in the study. It is relevant to mention that during the COVID-19 pandemic, university timetables were continuously adjusted according to local recommendations. Total population was divided into homogeneous strata, in accordance with year of study, then simple random probability sampling technique was implemented to reach a proportionate size out of each stratum. The total sample size was 188 (third-year students = 44, fourth-year students = 82, and fifth-year students = 62), and was calculated using a standard statistical formula, with a determined 95% confidence level, a 50% population proportion, a 5% margin of error, and a total population size of 350 (third-year students = 79, fourth-year students = 149, and fifth-year students = 122).

### Data acquisition

Data were collected using close ended structured questionnaires, delivered to students after obtaining verbal consents, with appropriate explanation of its content to eliminate chances of misinterpretation. Questionnaires were handed in-between classes when all students where inside the classroom, and filling was done by the students while collectors where available for answering queries. All data collectors were medical students, with no involvement of teachers, supervisors nor professors, in order to limit possibilities of involuntary student participation. Verbal consent was obtained from each student by data collectors by verbal communicating the purpose of the study and asking for their voluntary consent to participate prior to handing them the questionnaire and was documented separately to maintain anonymity.

The questionnaire was constructed by the authors using relevant background information, with parts being adopted, after modification, from the IPAQ, namely those pertaining to the WHO recommendation on minimum weekly hours. Simple face validation was performed prior to data collection. However, validity of the questionnaire was not tested further, and no further validation nor approval of the questionnaire were sought from national institutions.

#### The International PA Questionnaire (IPAQ)

Initial development of the IPAQ was commenced in Geneva in 1998, as an instrument for measurement of PA participation level^[10]^. This was followed by extensive reliability and validity testing, ultimately producing different versions that support its use in diverse contexts, including research incorporation^[57,58]^.

Although previous recommendations on IPAQ use suggested unaltered utilization, aiming toward prevention of misinterpretation bias, and although the IPAQ was not utilized, this study adopted a structured questionnaire that included the frequency of overall participation in PA, in addition to duration of each individual session rather than total number of weekly engagement hours, in order to further simplify surveying students’ responses. Thus, obtaining a broad understanding of general engagement of medical students, linked with their perspective.

### Data analysis

Statistical analysis was done using the SPSS software. Descriptive data were presented as categorical variables, including demographic, PA engagement and perspective variables, and presented as frequencies and percentages. Further cross-tabulation to determined relations of PA engagement with other variables was done using Chi-square testing with 95% confidence level, and a *P* value less than 0.05 to be considered as significant.

## Results

**Figure 1.**
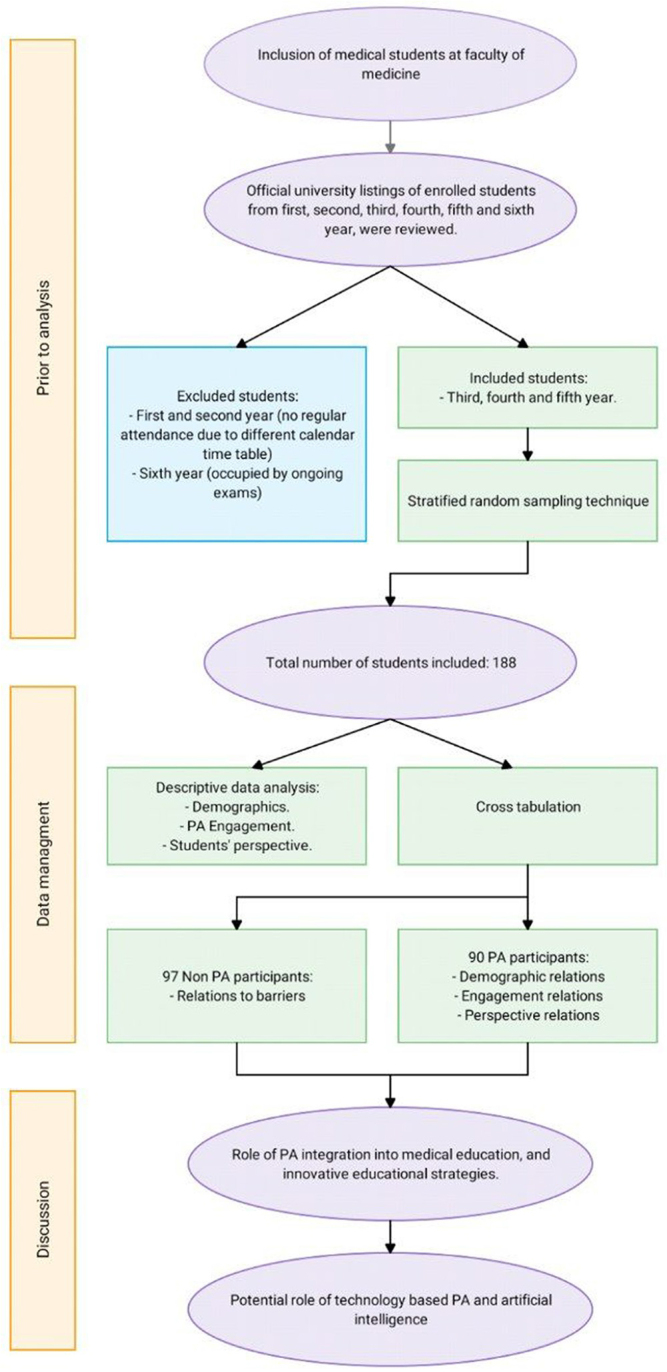
A flow diagram outlining medical students’ enrolment in the study outlining inclusion, exclusion, sampling, and methodology.

A total of 188 medical students were included in the study. All participants who were selected agreed to participate and verbally consented after appropriate questionnaire explanation by the authors. There were no missing data. A flow diagram outlining participants enrolment in the study is explained (Fig. 1).

### Demographics

Eighty-one (43.10%) participants were males, and 107 (56.90%) were females. Most students (136, 72.30%) were aged 20 to 25 years old, 44 (23.40%) were less than 20 years and 25 (4.30%) were older than 25 years of age (Table 1).

**Table 1:** Demographics and frequencies of physical activity engagement and perspective of medical students

Category	Variable	Total students (188)	
		Frequency	Percent (out of total)
Demographics			
Gender	Male	81	43.10%
	Female	107	56.90%
Age	<20 years	44	23.40%
	20-25 years	136	72.30%
	>25 years	8	4.30%
Engagement			
Participation in any planned PA	Yes	91	48.40%
	No	97	51.60%
Type of PA engaged	Group-based (sports, gyms, etc.)	54	28.72%
	Individualized (exercise, jogging, etc.)	37	19.68%
Frequency of planning	Regularly make plans	51	27.10%
	Rarely makes plans	40	21.30%
	Never plans	97	51.60%
Frequency of participation	Daily	14	7.40%
	Weekly	37	19.70%
	Monthly	40	21.30%
Duration of PA sessions	<30 minutes	26	13.83%
	30 minutes to one hour	41	21.81%
	More than one hour	24	12.77%
Motivation	To stay healthy	86	45.70%
	To interact socially	4	2.10%
	Physician’s advice	1	0.50%
Academics’ interference with PA	Yes	37	19.68%
	No	129	68.62%
	Sometimes	23	12.23%
Perspective			
PA promotes well-being and prevents non- communicable diseases	Agree	156	82.98%
	Disagree	32	17.02%
Barriers for PA engagement	Health/disability related	10	5.32%
	Academic	57	30.32%
	Environmental	39	20.75%
	No barriers	82	43.62%
PA promotion and advice	Limited to family and relatives	96	51.10%
	Extends to colleagues and friends	72	38.30%
	No active promotion	20	10.60%

### Medical students’ engagement in PA

Just over half of the students (51.60%) do not engage in PA, while the remainder does participate in planned PA. Of those, a portion engages in group-based activities, such as sports, while others practice individually. Some students regularly plan their PA, whereas others rarely do. When it comes to actual participation, a small percentage engages daily. Regarding the duration of each session, few students engage for less than 30 minutes. The primary motivator for participation in PA is promoting personal health, while a few students participate for social reasons or follow medical advice. Most students (68.62%) report that their academic workload does not interfere with their PA, though a minority experiences some level of interference (Table 1).

### Medical students’ perspective

Majority of students (82.98%) agreed that PA promotes well-being and helps prevent non-communicable diseases, while a smaller portion did not share this view. When it comes to potential barriers to PA, few students (20.75%) identified academic responsibilities as a hindrance, while others pointed to environmental factors. A small percentage cited disabilities or health issues as barriers (Table 1).

Just above half of the students promote participation in PA, while only a minority do not actively promote engagement in PA to others (Table 1).

### PA participation influences and relations

#### Demographic relations to PA participation

Out of those who participate in planned PA, 57.14% were males and 42.86% were females (*P* < 0.05). Most PA participants (73.63%) were in the 20 to 25 years age group (*P* < 0.05) (Table 2).

**Table 2:** Cross-tabulation and relations of physical activity participants and non-participants with different variables of demographics, engagement, and perspective of medical students

Category	Variable	Participation in any planned PA				P value
		Yes (91 student)		No (97 student)		
		Frequency	Percent (out of participants)	Frequency	Percent (out of non-participants)	
Demographics						
Gender	Male	52	57.14%	29	29.90%	0.00
	Female	39	42.86%	68	70.10%	
Age	<20 years	21	23.08%	23	23.71%	0.00
	20-25 years	67	73.63%	69	71.13%	
	>25 years	3	3.30%	5	5.15%	
Engagement						
Type of PA engaged	Group-based (sports, gyms, etc.)	54	59.34%	0	0%	0.00
	Individualized (exercise, jogging, etc.)	37	40.66%	0	0%	
Frequency of planning	Regularly make plans	51	56.04%	0	0%	0.00
	Rarely makes plans	40	43.96%	0	0%	
	Never plans	0	0.00%	97	100%	
Frequency of participation	Daily	14	15.38%	0	0%	0.00
	Weekly	37	40.66%	0	0%	
	Monthly	40	43.96%	0	0%	
Duration of PA sessions	<30 minutes	26	28.57%	0	0%	0.00
	30 minutes to one hour	41	45.05%	0	0%	
	More than one hour	24	26.37%	0	0%	
Motivation	To stay healthy	86	94.51%	0	0%	0.00
	To interact socially	4	4.40%	0	0%	
	Physician’s advice	1	1.10%%	0	0%	
Academics’ interference with PA	Yes	37	40.66%	0	0%	0.063
	No	32	35.16%	97	100%	
	Sometimes	23	25.27%	0	0%	
Perspective						
PA promotes well-being and prevents non-communicable diseases	Agree	82	90.11%	74	76.29%	0.028
	Disagree	9	9.89%	23	23.71%	
Barriers for PA engagement	Health/disability related	0	0.00%	10	10.31%	0.00
	Academic	7	7.69%	50	51.55%	
	Environmental	2	2.20%	37	38.14%	
	No barriers	82	90.11%	0	0%	
PA promotion and advice	Limited to family and relatives	50	54.95%	46	47.42%	0.21
	Extends to colleagues and friends	35	38.46%	37	38.14%	
	No active promotion	7	7.69%	13	13.40%	

#### PA participation relations to different aspects of engagement

More than half of the students who participate in PA (59.34%) lean toward group-based activities, such as sports and gym related activities. The rest practice individually by exercising and jogging (*P* < 0.05), half of them make regular plans (*P* < 0.05) and few students participate daily (*P* < 0.05). Most engage for less than one hour (*P* < 0.05). The majority (94.51%) reported health and well-being maintenance as the main motivator for their engagement in PA (*P* < 0.05) (Table 2). Additionally, Less than half of PA participants reported significant academic interference with their engagement in PA (*P* = 0.063) (Table 2).

#### PA participation relations to medical students’ perspective

Most of the students who participate in PA (90.11%), and most of those who do not (76.29%) agreed that PA promotes well-being and prevents non-communicable diseases (*P* < 0.05). However, all nonparticipants reported positively to the presence of potential barriers. Only a few (13.40%) PA participants did not actively promote participation in PA. In addition, about half of PA participants (54.95%), and less than half of PA nonparticipants (47.42%), had their promotion and advice activities limited to their family and relatives (*P* = 0.21) (Table 2).

## Discussion

### Medical students’ PA participation

The suboptimal degree of overall participation of medical students in PA demonstrated in this study reflects an outcome less than one would expect from future doctors^[13,14]^, despite the broad reach of information gathered to include all those who engage in any form, type and duration of PA. This finding has been demonstrated in the past, as sedentary behavior was prominent in similar populations^[30,32]^. Moreover, the drastic impact of the corona virus diseases (COVID-19) pandemic further escalated lack of PA participation, as demonstrated recently in a study involving Italian medical students^[30]^.

Furthermore, demographic findings suggest that males are more expected to engage in PA (*P* < 0.05) (Table 2). However, previous evidence, from a Canadian study, found that female medical students are more motivated to promote and advocate for PA participation^[25]^.

The relatively low frequencies of daily PA engagement (14 out of 91, 15.38%), with a minimum of one hour per session (24 out of 91, 26.37%) amongst PA participants, suggest that less than one tenth accommodate the minimum WHO PA guidelines recommendation. Though the traditional IPAC was not utilized unaltered. Contrary to this finding, a recent study in Saudi Arabia demonstrated significantly higher proportions achieving WHO recommendations, despite the prominence of sedentary behavior^[40]^. This answers one of the main objectives of the study, as students’ level of engagement in PA in not significantly high, and is closely similar to a number of finding reported in the past.

Furthermore, the relative tendency to adopt a group-based approach when engaging in PA signifies the importance of social interactions as a potential influencer, despite lack of responses expressing socialization as a possible motivator (*P* < 0.05%) (Table 2). Moreover, targeted utilization of social media, which has significantly impacted young adults, was found by a Serbian study to increase the likelihood of sufficient PA participation^[59]^, in addition to quality of life enhancement when used to facilitate combined exercise interventions^[60]^.

Medical students’ engagement in PA does not seem to be significantly interfered by their academic responsibilities. These further complements previous findings demonstrated by an Indonesian study highlighting the positive influence of PA on burnout stress outcomes associated with the relatively high academic load in medical education^[28,33]^.

### Medical students’ perspective

The acceptable degree of understanding of PA related health benefits, and preventive roles, demonstrated in this study, reflects an adequacy of background knowledge resembling that of recent findings from a scoping review on medical schools from Singapore and the UK^[24]^. However, a quarter of those who do not participate also do not think PA is useful in disease prevention, suggesting potential rejection of an evidence-based approach.

A consequently expected action, therefore, is advocacy through active promotion, though knowledge does not always equate to action. However, its apparent limitation within the zone of family and relatives, may be explained by the relatively focused social interactions. Moreover, recent evidence from a Canadian study suggested that implemented measures aiming for more PA involvement impacts students’ promotional attitude^[22]^. The effect of active PA participation on active PA promotion is therefore apparent, yet not significantly high. Converse, wider advocation may also positively affects future practice of PA prescription. A previous survey highlighted residents’ expression of their desire for training in exercise prescription, despite adequate background knowledge, which sheds light on the significance of systematic curricular development^[42]^. In support of this evidence, even among students, in this study, who do not participate in PA, few do advocate to others, reflecting that advocacy and promotion could potentially be independent from active PA engagement. Therefore, linking PA engagement to students’ perspectives in this study provided additional understanding of the absence of dependence on both.

The unique considerations of undergraduate medical education that are derived from a relatively bulky academic load, including preclinical and clinical studies, makes it perceivable as a potential barrier from sufficient PA participation, as found in this, and previous studies. However, the level of resilience demonstrated by medical students in the past, and continues to be, acts as a buffering utility by which they succeed in adaptation and accommodation^[61]^.

### Impactful innovation in medical education

Recent enlightenment of medical institutions on the need for contemporary increments in PA engagement amongst undergraduate medical students, lead to the development of novel educational strategies aiming to achieve this goal, such as embedding of PA within the curriculum of several medical schools^[49-53]^, including positively impact on students’ fitness, found by a Colombian study^[16]^.

Recent movements for PA integration in undergraduate medical education curriculum were adopted at an authoritative level, such as recommendations commissioned by Public Health England^[50,52]^. As significant portion of students seen in this study did not consider their academic load a potential barrier for PA engagement, this recent movement may smoothly integrate in future developments. Moreover, a recent Delphi study explored the feasibility of integrating an exercise medicine syllabus into undergraduate medical schools’ curriculum and found a promising level of acceptance in the UK^[53]^. It is relevant to mention that the university where the study took place organizes occasional competitive sport events where students could voluntarily participate. However, no official curricular PA integration where implemented.

On a broader level, the American Medical Society for Sports Medicine advocated for the development of core curricula for both US medical schools and residency programs, which was endorsed by the Canadian Academy of Sport and Exercise Medicine, in an effort to enhance PA engagement and promotion within medical education^[54]^.

Furthermore, strategic utilization of newly designed mentoring programs, as well as monitoring and surveillance integrated tools, further compliments educational strategies. A recent survey from Taiwan evaluated a redesigned clinical mentoring program launched in a medical Center, incorporating mentor qualifications, enhancers for mentee relationship with mentors, performance evaluation, and incentives, and was found to significantly improved overall performance^[62]^. Its implementation for PA engagement, however, warrants further assessment.

Additionally, a mixed methods study in Thailand introduced, and assessed a newly developed tool, the Medical School PA Report Card, and highlighted the need for an improved environmental quality in order to establish PA promotional policies^[63]^. Furthermore, a recently developed learning objectives for physician education in PA counselling and prescription, were implemented in several Canadian medical schools in order to enhance medical students’ promotional attitude^[55]^. On the other hand, a recently developed e-learning resource (MEdic GAming, MEGA) was qualitatively evaluated in a UK medical school in order to understand medical students’ perspective. However, they felt the need for an approach blended with face-to-face PA learning^[64]^.

### Technology and AI-driven PA incorporation

Technology-based PA interventions were demonstrated in the past to possess significant PA promotional value. Among them, active video games, VR, smart mobile phone applications, social media and videoconferencing were shown to increase the PA level of participation, as well as mental health^[64]^. Recent evidence suggested beneficial outcomes when they are offered to both patients post bariatric surgery, and those with chronic disease^[65,66]^.

Moreover, mobile applications, internet-based platforms and devices, such as activity bracelets, constitute essential behavioral strategies including goal marking, self-monitoring and self-directed feedback^[67]^. A recent systematic review highlighted a specific incremental impact of smartphone applications on PA engagement, which is considered an accessible and feasible utility^[68]^. This sheds light on the effect of access to such facilities, with may be accessible to more financially capable students, on the level of PA engagement.

Newer technology-based intervention, such as VR incorporated bike exercise was recently assessed among college students in comparison with traditional utility. It was found to be a potentially effective, enjoyable, and motivating PA tool^[69]^. Though further diverse evaluation of its efficacy is still warranted.

AI is an innovative technology that is rapidly transforming the medical education landscape. AI can evaluate curriculum effectiveness and assist students in receiving tailored learning content enhanced by their feedback, in addition to support provision for educators^[70]^. However, the possibility of error alongside its relatively high cost and the need for special training on its use, may hinder wider use amongst medical institutions.

The current application of AI in medical education is focused on clinical specialty training. Furthermore, evidence suggests that medical students have increased interest in addition of AI related courses to the medical curriculum^[47]^. This sheds light on the possibility of AI-driven PA tools integration into the medical curriculum as a potential participation and an enhancer for PA promotion. Thus, further studies on this particular role are warranted.

### Limitations

The exclusion of first- and sixth-year students from study, may have hindered production of results representative of the intended population, as comparison was not performed between pre-clinical and clinical medical students. Moreover, the short duration of the study, and the need for a larger sample study may have affected power of the study. Furthermore, utilization of a structured questionnaire, which was constructed by the author, instead of the validated unaltered form of the IPAQ during data acquisition, though outside the scope of the study, alongside lack of additional validation and testing of the utilized questionnaire, may have concealed additional statistically significant outcomes, in addition to limiting the possibility of generating a more specific targeted conclusion. Additionally, this study utilized statistical analysis techniques confined to descriptive analysis and chi-square tests, which may have limited the strength of the study. Therefore, future studies should utilize them to assess level, degree and promotional attitude of PA participation amongst medical students, with additional focus on when they use technology-based and AI-driven PA instruments.

## Conclusion

The acceptable background knowledge of medical students and understanding of promotional and preventive significance of PA, does not necessarily equate to an applaudable level of engagement. Moreover, medical students agree on potential positive benefits of PA, yet their promotional advocacy involve a more focused environment.

Previous evaluations suggest that systematic integration of PA into medical education curricula, bearing in mind contextual diversity, may positively impact their level of participation. Future studies should assess innovative strategies, such as targeted mentoring programs, PA report cards and exercise prescription training in the context of medical education. Additionally, comparative studies should be considered to differential level of PA engagement and promotion in institutions with PA integrated curriculum.

Furthermore, future studies should also evaluate the influence of technology-based and AI-driven PA on medical students’ PA engagement, focusing on feasible utilities such as smart phones, as it has shown promising results in different populations.

## Data Availability

The datasets generated during and/or analyzed during the current study are available from the corresponding author on reasonable request.

## Research registration unique identifying number (UIN)

UIN researchregistry10728.
